# Nuclear YAP localization as a key regulator of podocyte function

**DOI:** 10.1038/s41419-018-0878-1

**Published:** 2018-08-28

**Authors:** Jakob Bonse, Dirk Oliver Wennmann, Joachim Kremerskothen, Thomas Weide, Ulf Michgehl, Hermann Pavenstädt, Beate Vollenbröker

**Affiliations:** Department of Nephrology, Internal Medicine D, Hypertension and Rheumatology, University Hospital Muenster, Muenster, Germany

## Abstract

Podocytes are crucial for the establishment of the blood-urine filtration barrier in the glomeruli of the kidney. These cells are mainly affected during glomerulopathies causing proteinuria and kidney function impairment. Ongoing podocyte injury leads to podocyte loss, finally followed by end-stage kidney disease. Podocytes display a predominant nuclear localization of YAP (Yes-associated protein), one effector protein of the Hippo pathway, which regulates the balance between proliferation, differentiation, and apoptosis in cells. Nuclear active YAP seems to be critical for podocyte survival in vivo and in vitro. We can show here that different treatments leading to sequestration of YAP into the cytoplasm in podocytes, like decreased rigidity of the substrate, incubation with dasatinib, or overexpression of Hippo pathway members result in the induction of apoptosis. A RNA sequencing analysis of large tumor suppressor kinase 2 (LATS2) overexpressing podocytes confirmed a significant upregulation of apoptotic genes. The downregulation of Hippo pathway components suggests a feedback mechanism in podocytes. Noteworthy was the regulation of genes involved in cell–cell junction, the composition of the extracellular space, and cell migration. This suggests an influence of Hippo pathway activity on podocyte integrity. As focal segmental glomerulopathy (FSGS) goes along with an activation of the Hippo pathway in podocytes, a comparison of our data with two independent studies of transcriptional regulation in human FSGS glomeruli obtained from the Nephroseq database was performed. This comparison affirmed a multitude of consistent transcriptional changes concerning the regulation of genes influencing apoptosis and the Hippo signaling pathway as well as cell junction formation and cell migration. The link between Hippo pathway activation in podocytes and the regulation of junction and migration processes in vivo might be a fundamental mechanism of glomerular sclerosis and loss of renal function.

## Introduction

Glomerulopathies are the main reason for end-stage renal disease. In nearly all glomerulopathies, podocytes are directly or indirectly damaged^1^. Podocytes together with the glomerular basal membrane (GBM) and the endothelium form the blood-urine filtration barrier in the glomeruli of the kidney. With their large foot processes, interdigitating with the neighboring cells, they surround the glomerular capillaries in a tight cell layer, holding contact to the GBM. The slit diaphragm between the foot processes allows free filtration of water, electrolytes, and small molecules, but prevents the loss of bigger proteins into the urine^2^. Injury of the podocyte or the slit diaphragm leads to proteinuria and, frequently, to podocyte loss^3^. As podocytes are postmitotic cells, their loss can merely be compensated by hypertrophy of neighboring cells. Insufficient compensation results in loss of kidney function.

In contrast to mitotic active renal epithelial cells, podocytes display a predominant nuclear localization of YAP (Yes-associated protein), one effector protein of the Hippo signaling pathway^4–6^. The Hippo pathway is a kinase cascade known to regulate the balance between proliferation, differentiation, and apoptosis in cells. Core components of the mammalian Hippo pathway are the kinases mammalian STE20-like protein kinase 1 and 2 and the large tumor suppressor kinases 1 and 2 (LATS1/2)^7^. Phosphorylation and activation of LATS 1/2 finally leads to phosphorylation of YAP and its paralog transcriptional co-activator with PDZ-binding motif (TAZ). Both are thereby excluded from the nucleus, affecting their ability to act as co-transcription factors for the transcription factor family of transcription enhancer factor TEF1-4, which influences the expression of a wide range of genes involved in cell proliferation, survival, and migration.

We could previously show that the Hippo pathway in cultured podocytes is largely inactive due to low LATS activity and that the resulting nuclear localization of YAP is critical for podocyte survival. Induction of the Hippo pathway, e.g., through overexpression of the upstream component and LATS activator WW and C2 domain containing 1 (WWC1/KIBRA) leads to apoptosis of podocytes in vitro^6^. In mice, podocyte-specific YAP deletion results in podocyte depletion, proteinuria, and focal segmental glomerulosclerosis (FSGS)^5^. Human FSGS patients show an increased phosphorylation of YAP (p-YAP), which is equivalent to YAP inactivation^8^. However, the changes in podocyte gene transcription after Hippo signaling activation and the role of LATS kinases in this process remained unclear.

In this report, we show that Hippo pathway activation in podocytes through several treatments leads to apoptosis. A transcriptome analysis of podocytes overexpressing LATS2 reveals changes in the expression profiles of known YAP target genes as well as the activation of so far unknown signaling pathways. A comparison with the gene regulation from human FSGS glomeruli gives new insights into Hippo signaling-mediated gene regulation in podocytes during disease.

## Materials and methods

### Cell transfection, lysate preparation, and western blot analysis

Western blot analysis was done as described before^6^. Summarized, cells were treated with ice-cold lysis buffer (20 mM Tris-HCl pH 7.4, 20 mM NaCl, 1 mM EDTA, 50 mM NaF, 10 mM Na_4_P_2_O_7_, 1% Triton X-100) containing protease (Roche, Basel, CH) and phosphatase (Sigma-Aldrich, Darmstadt, GER) inhibitors and centrifuged at 14,000×*g* for 10 min. The supernatants were transferred to 2× SDS sample buffer (20% glycerin, 125 mM Tris-HCl pH 6.8, 10% SDS, 0.2% bromphenol blue, 5% β-mercaptoethanol) and incubated at 95 °C for 5 min. Afterwards, the lysates were pushed through a 20-gauge needle, and equal volumes of cell lysates were separated on 8–15% SDS-PAGE gels (Bio-Rad, Munich, GER). Proteins were transferred to a PVDF membrane (Merck Millipore, Darmstadt, Germany) and incubated for 1 h at room temperature in blocking buffer (5% BSA powder dissolved in TBS containing 0.05% Tween-20 (TBS-T)). The amount of proteins was marked by using β-tubulin (Sigma) or GAPDH (Covance, Princeton, NJ, USA) as loading controls. The quantification of western blot signals was done as described before^6^. For the detection of the specific proteins, following antibodies were used: YAP (D8H1X) XP #14074; cleaved caspase 3 #9664; p-LATS1 #8654; LATS2 #5888; p-YAP #13008P; PARP #9542; cleaved PARP #5625; caspase 3 #9665; caspase 9 #9508; cleaved caspase 9 #7237, caspase 7 #12827; cleaved caspase 7 #8438 (Cell Signaling Technology, Danvers, MA, USA).

### Cell culture

Human immortalized podocytes (AB8/13) (kindly provided by M. Saleem) and HEK293T (Thermo Fisher Scientific, Milan, Italy) were cultivated as described earlier^6^. For transient transfection, HEK293T were transfected by the calcium phosphate method as described earlier^9^. In order to determine the influences of soft matrices, podocytes were cultured for 24 h on glass bottom plates with attached collagen IV-coated hydrogels containing surface rigidities of 1, 12, 25, and 50 kPa (Matrigen Life Technologies, Brea, CA, USA).

### Immunofluorescence analysis

AB8/13 podocytes and sections from rat kidneys were used for indirect immunofluorescence analysis. A detailed description is published before^6^. Cells were treated with Dasatinib (Sigma, CDS023389) solved in DMSO in a concentration of 500 nM for 48 h. For the detection of proteins and cell compartments, following agents were used: YAP sc-101199, WT1 sc-192 (Santa Cruz Biotechnology, Dallas, Texas, USA); cleaved Caspase 3 #9664 (Cell Signaling); ANTI-FLAG^®^ M2 F3165 (Sigma); fluorescence-labeled secondary antibodies and Phalloidin (Alexa Fluor^®^), 4′,6-diamidino-2-phenylindole (DAPI) (Invitrogen, Thermo Fisher).

### Immunohistochemistry analysis

In brief, paraffin-embedded kidney sections (5 μm) were treated with 0.01 M citric acid buffer (pH 6.0, boiled for 3 min) for antigen retrieval. Sections were incubated overnight with a mouse antibody (1:100 in 1% BSA) against YAP (Santa Cruz, sc-101199). Afterwards, sections were incubated with a biotinylated secondary antibody against mouse (1:200, Vector Laboratories, Burlingame, CA, USA) in PBS. Finally, the sections were stained by incubation with avidin-biotin peroxidase (Vector Laboratories) and reaction with DAB. Hemalum was used for nuclear staining. The rat kidney sections were from healthy, untreated 8–10-week-old male Sprague–Dawley rats (Charles River, Germany). The human kidney section shown was from a patient with interstitial nephritis of a middle-aged man, provided by the Institute for Pathology, University Hospital Muenster, Germany. The experiments with human material were approved by the local ethics commission (2016-616-f-S) and the written consent was obtained from all patients.

### Cell viability assay

The RealTime-Glo™ MT Cell Viability Assay (Promega, Dübendorf, Switzerland) was used according to the manufacturer’s instructions to measure the cell viability at real time. In white bottom 96-well plates (Nunclon, Thermo Fisher), 1000 podocytes were seeded in 50 µL medium per well for 24 h, conditionally induced with 125 ng/mL doxycycline and treated with dasatinib (50 nM-5 µm) or DMSO (0.1%) as solvent control. To start the reaction, 50 µL of a 1× RealTime-Glo™ enzyme-substrate-mix diluted in medium were added to the cells. The measurement at indicated time points started after 10 min incubation using a microplate reader (TECAN).

### RNA isolation and quantitative real-time PCR

Total RNA was isolated from cell culture podocyte samples using the GenElute Mammalian total RNA Miniprep Kit (Sigma-Aldrich) according to the manufacturer’s instructions. Complementary DNA was synthesized using the Superscript III Reverse Transcriptase kit (Invitrogen). Quantitative real-time PCR (qPCR) based on SYBR Green (Applied Biosystems, Foster City, CA, USA) dye was performed by the Core Facility Genomics (Medical Faculty, University Hospital Muenster, Germany) using CFX384 Touch (Bio-Rad, Hercules, CA, USA). Relative gene expressions were normalized to GAPDH and compared using the comparative ΔCT method^10^.

### Generation of stable cell lines

Generation of stable cell lines is described in detail before^9,11^. We generated stable, doxycycline-inducible podocytes that overexpress LATS2 WT, LATS T1041E, LATS T1041A, YAP WT, or YAP S127A, and cells that overexpress short hairpin RNA against YAP using the established lentiviral pINDUCER system^11,41^. The following 97-mer linkers were used to create RNAi cell lines:

YAP sh1: 5′–TGCTGTTGACAGTGAGCGCCACATCGATCAGACAACAACATAGTGAAGCCACAGATGTATGTTGTTGTCTGATCGATGTGATGCCTACTGCCTCGGA–3′;

YAP sh2: 5′–TGCTGTTGACAGTGAGCGCTGAGAACAATGACGACCAATATAGTGAAGCCACAGATGTATATTGGTCGTCATTGTTCTCAATGCCTACTGCCTCGGA–3′.

### Flow cytometric analysis with FITC-Annexin V and propidium iodide

Medium was removed and cells were detached by Accutase (Thermo Fisher) treatment for 5 min. After washing with PBS, cells were centrifuged (5 min, 1200 r.p.m.) and re-suspended in PBS with cations, 0.5% fetal calve serum (FCS), and 0.05% NaN_3_. Cells were stained for 20 min with Fluorescein isothiocyanate (FITC)-conjugated Annexin V and propidium iodide (PI) to identify apoptotic cells by exposed phosphatidylserine and to distinguish necrotic cells. Early apoptotic, Annexin V-positive and PI-negative cells were determined by fluorescence-activated cell sorting (FACS) with a BD FACSCalibur™ analyzer and analyzed with BD CellQuest™ Pro Software. The number of apoptotic cells in samples is displayed normalized to non-induced control.

### RNA sequencing

In order to determine the changes in the transcriptome of podocytes with and without doxycycline treatment, the RNA of four samples from each condition was isolated and analyzed by RNA sequencing. Therefore, library preparation of the total RNA was performed with the NEBNext Ultra RNA Library Prep Kit for Illumina (New England BioLabs, Ipswich, MA, USA) and NEBNext Poly(A) mRNA Magnetic Isolation Module (New England BioLabs). Single-read sequencing was performed using a NextSeq^®^ 500 System (Illumina) with a depth of ~400 million single reads and a read length of 80 bp. Using a molecular barcode, the samples were demultiplexed (bcl2fastq2) to fastq data and quality controlled (FastQC). Trimmomatic (v0.33)^12^ was used for adapter trimming and read filtering. The resulting reads were aligned to the human reference genome (Homo sapiens HG19) using TopHat^13^. The aligned reads were assembled into transcripts in means of FPKM values (fragments per kilobase of exon per million fragments mapped), and differentially expressed genes (DEGs) and transcripts were reported using the Cufflinks package^14^. To evaluate the result fidelity, the dynamic range and distribution of the FPKM values across the samples could be examined and displayed with boxplots and the relationships between the conditions were analyzed by dimensional reduction in a multi-dimensional scaling using CummeRbund^15^. To organize the genomic data, the DEGs were clustered in means of regulation patterns and visualized by a hexagonal map using supraHex^16^. Differentially expressed genes could be compared to gene expression data obtained from Nephroseq (www.nephroseq.org; Nephroseq Classic (v4), University of Michigan, Ann Arbor, MI) and visualized using Venn diagrams from Ghent University (http://bioinformatics.psb.ugent.be/webtools/Venn/; VIB/UGent, Gent, Belgium).

### Quantification and statistical analysis

Signals derived from immunoblot were densitometrically quantified using ImageJ (http://rsbweb.nih.gov/ij/). Graphing and statistical analysis (unpaired student’s *t*-test or Mann–Whitney-*U*) were performed using the GraphPad Prism 5 software: NS: *P* > 0.05; *P ≤ 0.05; **P ≤ 0.005; ***P ≤ 0.001.

## Results

### The rigidity of the extracellular matrix controls YAP localization in podocytes

**Fig. 1:**
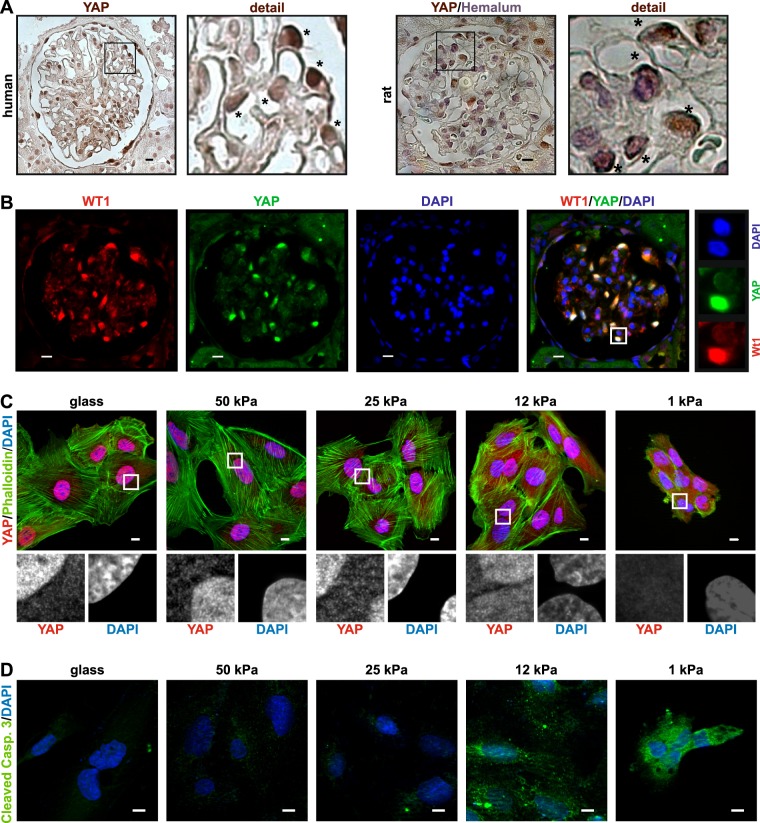
Podocyte YAP localization is controlled by matrix rigidity. **a** IHC-staining of YAP from a representative glomerulus in human and rat kidney sections with details showing predominant nuclear localization of YAP. Nuclei in rat kidney section was visualized with hemalum. **b** Co-immunofluorescence staining of a rat glomerulus revealed co-localization of nuclear YAP with the nuclear podocyte marker WT1. **c** Co-immunofluorescence staining of YAP, DAPI, and phalloidin, or **d** cleaved caspase 3 (CC3) in cultured human podocytes on different surface rigidity (glass, 50/25/12/1 kPa). Scale bars = 10 µm

The nuclear localization of YAP in podocytes in vivo is still a matter of discussion and seems to be regulated tightly^5,17^. Our observations confirmed an enhanced nuclear expression of YAP in peripheral cells of the glomerulus, most probably podocytes, in human as well as rat kidney sections (Fig. 1a). A co-staining of YAP with the podocyte nuclear marker protein Wilms tumor 1 emphasizes the predominant nuclear YAP localization in podocytes (Fig. 1b). Because of this, we assume that Hippo signaling is off and YAP is able to enter the nucleus. Since Hippo signaling is known to be activated by a change of the rigidity of the extracellular matrix (ECM)^18^, we examined the YAP localization in podocytes cultured on soft matrices with elastic moduli comparable to in vivo tissues (1–50 kPa; Fig. 1c). The in vivo ECM rigidity of the GBM as the attachment surface of podocytes is unknown, but is likely within the range of 1–50 kPa^19^. On very soft matrices (≤12 kPa), podocytes displayed an aggregated morphology with enhanced YAP cytoplasmic localization (Fig. 1c). Especially the attachment of the cells to the 1 kPa stiff ECM seemed to be exacerbated, resulting in a reduction of actin stress fibers and a loss of cells. Staining of cleaved caspase 3 revealed that the loss of nuclear YAP in podocytes cultured on very soft matrices (≤12 kPa) is accompanied with the induction of apoptotic processes (Fig. 1d). Forty-eight hours after seeding, nearly no cells were visible on the matrices with the rigidity of 1 or 12 kPa (data not shown). This suggests that the rigidity of the ECM is important for regulation of Hippo signaling in podocytes and that nuclear YAP is essential for podocyte surveillance. Therefore, culturing podocyte on matrices harder than 12 kPa is inevitable.

### Nuclear YAP regulates podocyte cell viability

Obviously, a change of the ECM rigidity as well as an activation of the Hippo pathway by overexpression of the pathway member KIBRA leads to podocyte apoptosis^6^. To test an additional Hippo pathway inducer, we used dasatinib, an FDA-approved drug for the therapy of leukemia^20^. The use of dasatinib is associated with proteinuria and nephrotic syndrome as adverse effect^22–24^. Dasatinib is a potent inhibitor of receptor tyrosine kinases like SRC family kinases and the BCR-ABL fusion protein^24^. Although it will therefore have a lot of additional effects, dasatinib was recently identified as a small molecule agent, which inhibits the nuclear localization of YAP and triggers its phosphorylation in breast cancer cells^25^.

**Fig. 2:**
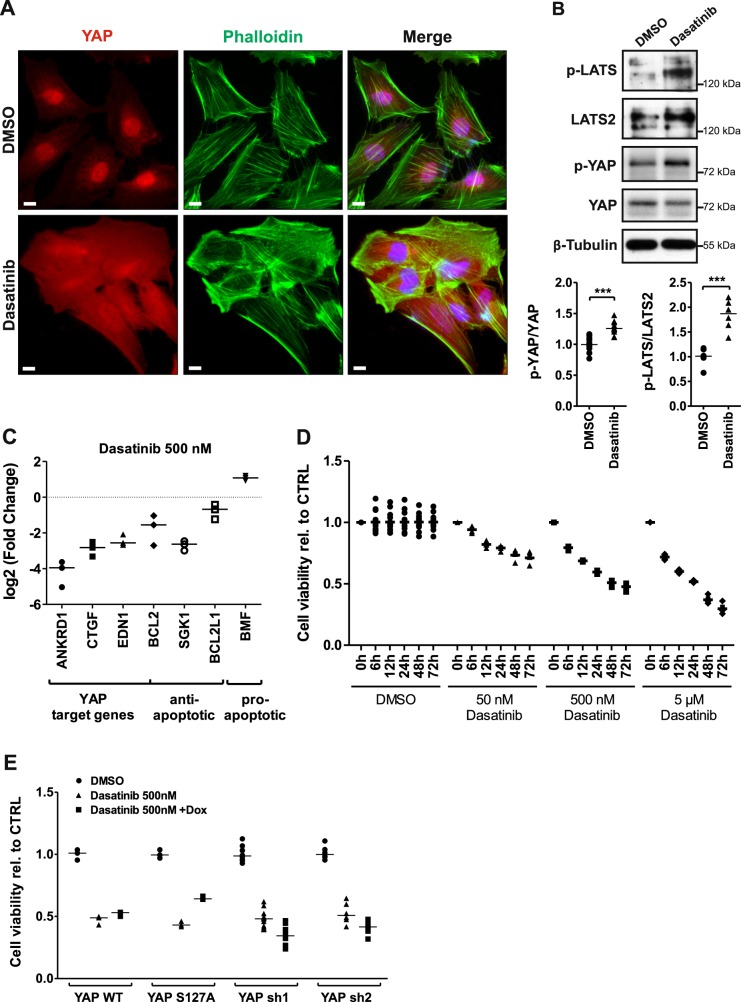
Activation of Hippo pathway leads to a YAP-dependent reduction of podocyte cell viability. **a** YAP, phalloidin, and DAPI co-staining of dasatinib (500 nM, 48 h)-treated cultured human podocytes. Scale bars = 10 µm. **b** Phosphorylation of LATS and YAP monitored via western blot in protein lysates from dasatinib-treated podocytes (500 nM, 48 h) with densitometric quantification of p-YAP/YAP (*N* = 10) and p-LATS/LATS2 (*N* = 6). **c** Quantitative PCR analyses of YAP target genes as well as anti-/pro-apoptotic genes (*N* = 3). **d** Cell viability determination of dasatinib-treated human podocytes with a luciferase-based luminescence assay, concentration, and duration as indicated. Data are relative to control and conditions at 0 h, DMSO serves as vehicle control. **e** Cell viability determination of stable doxycycline-inducible podocyte cell lines overexpressing YAP wild-type (YAP WT, *N* = 3), permanent active YAP (YAP S127A, *N* = 3), or two different YAP knockdown hairpins (YAP sh1, *N* = 12; YAP sh2, *N* = 6) after dasatinib treatment. DMSO served as vehicle control. ***P ≤ 0,001, student’s *t*-test

Treatment of cultured podocytes with dasatinib (500 nM, 48 h) led to a nuclear export of YAP and a disturbance of actin stress fibers (Fig. 2a). Hippo pathway activation was indicated by enhanced phosphorylation of LATS and its target YAP (Fig. 2b). Consequently, the loss of nuclear YAP resulted in a significantly reduced expression of the YAP target genes ankyrin repeat domain 1, connective tissue growth factor, endothelin 1, and B cell lymphoma 2. Furthermore, decreased transcription of the anti-apoptotic genes B cell lymphoma 2, serum/glucocorticoid regulated kinase 1 and BCL2-like 1 as well as the increased transcription of the apoptotic activator gene BCL2 modifying factor were detected indicating an enhanced apoptosis of the dasatinib-treated cells (Fig. 2c).

Dasatinib treatment reduced podocyte cell viability in a time and dose-dependent manner (Fig. 2d). Additional experiments with different inducible podocyte cell lines revealed that overexpression of YAP wild-type (YAP WT) or a non-phosphorylatable, constitutively active YAP mutant (YAP S127A) rescues the effect of dasatinib on cell viability. On the other hand, a siRNA-mediated YAP knockdown by two different short hairpins (YAP sh1, YAP sh2) potentiates the dasatinib effect (Fig. 2e).

### LATS2 phosphorylation at T1041 regulates podocyte viability and induction of apoptosis

**Fig. 3:**
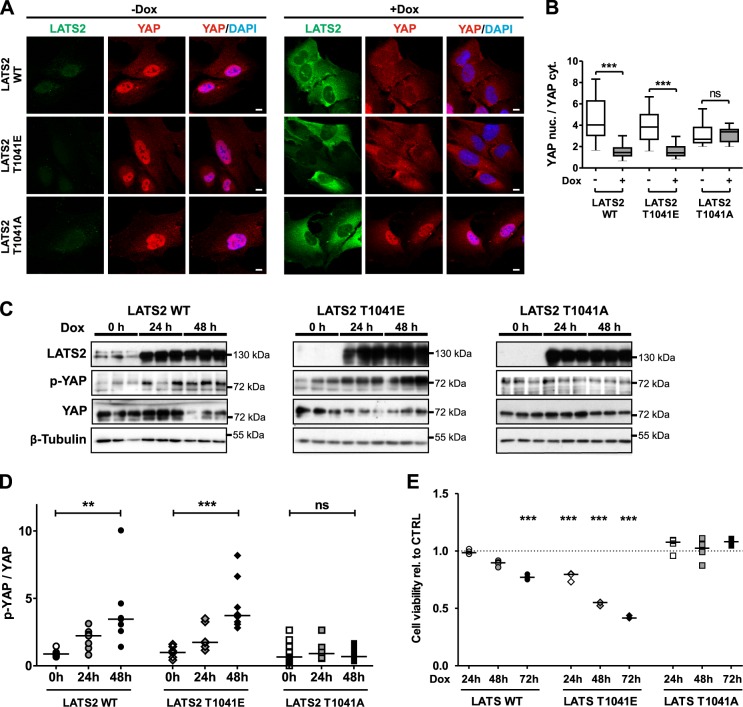
Loss of cell viability due to LATS2 overexpression depends on phosphorylation site T1041. **a** Representative co-immunofluorescence staining of LATS (FLAG/green), YAP (red), and DAPI (blue) in untreated or doxycycline-treated inducible stable human podocyte cells overexpressing LATS2 wild type (LATS2 WT), permanently active (LATS2 T1041E), or permanently inactive LATS2 (LATS2 T1041A). Scale bars = 10 µm. **b** Quantification of the nuclear YAP (YAP nuc.) to cytoplasmic YAP (YAP cyt.) intensity ratio of cells from A (WT, *N* = 39; T1041E, *N* = 53; T1041A, *N* = 21). ns: *P* > 0.05; ***P ≤ 0.001, student’s *t*-test. **c** Protein lysates from LATS2-overexpressing cell lines after indicated time points of doxycycline induction monitored by western blot to determine LATS2 and YAP protein levels and YAP phosphorylation. β–Tubulin served as loading control. Triplicates of each time point. **d** Densitometric quantification of YAP phosphorylation in C (WT, *N* = 7; T1041E, *N* = 7; T1041A, *N* = 9). ns: *P* > 0.05; **P ≤ 0.01; ***P ≤ 0.001, Kruskal–Wallis test. **e** Cell viability of LATS2-overexpressing cell lines was determined by luciferase-based luminescence assays at indicated time points after induction relative to untreated cells (WT, *N* = 4; T1041E, *N* = 3; T1041A, *N* = 4). ***P ≤ 0.001, one-way ANOVA

**Fig. 4:**
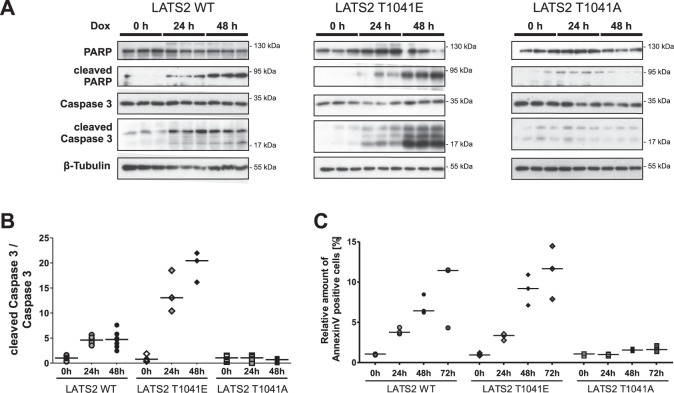
LATS2 overexpression-induced apoptosis depends on T1041 phosphorylation site. **a** Triplicates of protein lysates from LATS2 wild-type (LATS2 WT), permanently active (LATS2 T1041E), or permanently inactive LATS2 (LATS2 T1041A) overexpressing cell lines at indicated time points after doxycycline induction monitored on western blot to determine cleavage of apoptotic marker proteins poly (ADP-ribose) polymerase (PARP) and caspase 3. β–Tubulin served as loading control. **b** Densitometric quantification of cleaved caspase 3 relative to caspase 3 protein level in A (WT, *N* = 7; T1041E, *N* = 3; T1041A, *N* = 6). **c** Flow cytometric analysis with FITC-Annexin V and propidium iodide performed to detect apoptotic cells at indicated time points after induction of LATS2 wild-type, permanently active, or permanently inactive LATS2 overexpression (*N* = 3)

YAP translocation into the cytoplasm as a response to Hippo pathway activation depends on the LATS kinase activity that needs phosphorylation of Threonine 1041^26^. We could confirm this for podocytes using inducible, stable human podocyte cell lines overexpressing LATS2 wild-type (LATS2 WT), a permanently active mutant of LATS2 (LATS2 T1041E), or a permanently inactive mutant of LATS2 (LATS2 T1041A). Overexpression of LATS2 WT and LATS2 T1041E but not of LATS2 T1041A leads to nuclear export of YAP (Fig. 3a, b). Consistently, only active LATS variants lead to YAP phosphorylation and subsequent lower cellular YAP concentration due to enhanced degradation (Fig. 3c, d). An analysis of the cell viability displayed that the induction of LATS2 WT expression reduces podocyte viability in a time-dependent manner. This effect was strengthened by induction of LATS2 T1041E expression, but was completely absent after overexpression of the inactive variant LATS2 T1041A (Fig. 3e). An increased cleavage of procaspase-3, -7, and -9 to their active subunits as well as of PARP (poly (ADP-ribose) polymerase) proved the induction of apoptosis after LATS activation (Fig. 4a, b and Supplementary Fig. 1). Cleavage of initiator caspase-9 may indicate that activation of Hippo signaling induces the intrinsic apoptosis pathway. FACS analysis on Annexin V and PI-stained podocytes also revealed increasing amounts of cells in early stages of apoptosis due to overexpression of active LATS (Fig. 4c).

### Transcriptional analysis of Hippo pathway activation in podocytes

**Fig. 5:**
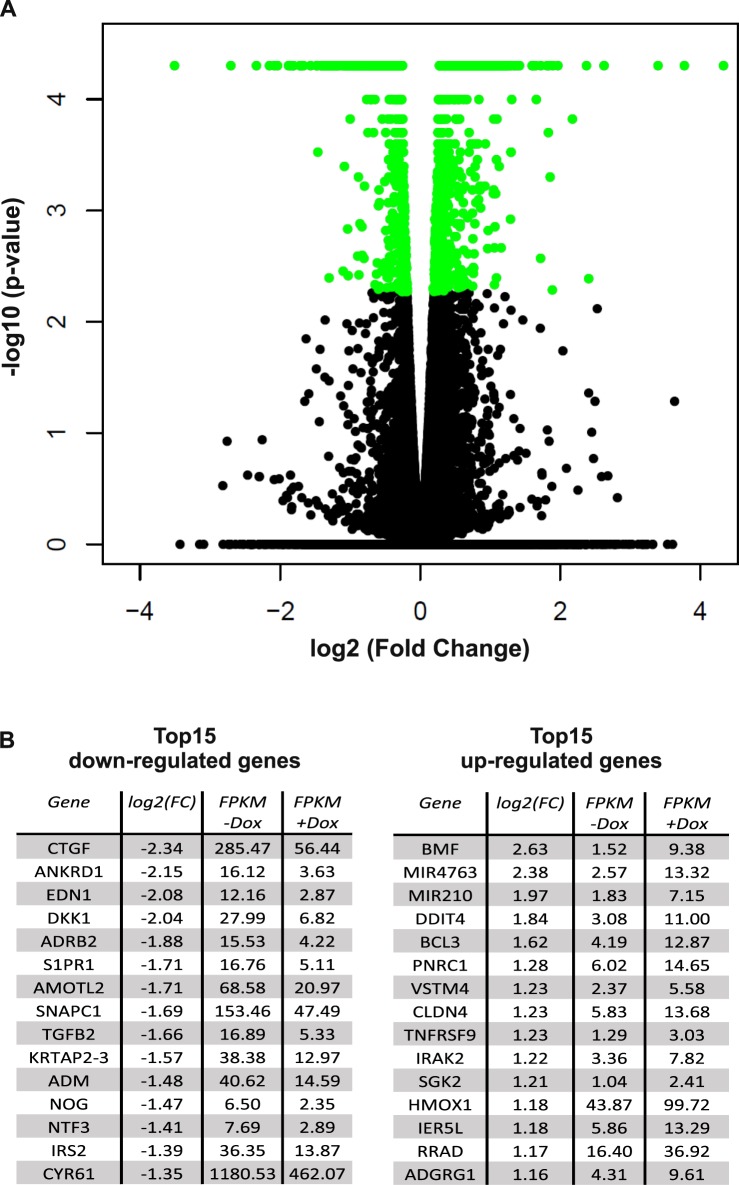
RNA sequencing analysis after Hippo pathway activation by LATS2 wild-type overexpression in human podocytes RNA sequencing analysis of 8 h doxycycline-induced human podocytes overexpressing LAST2 wild-type vs. untreated cells. **a** Volcano plot comparing negative (base 10) logarithmic *P*-value (*y* axis) to (base 2) logarithmic fold change (*x* axis) of all 25,459 detected genes. A total of 1428 transcriptionally changed genes with false discovery rate (FDR) corrected *P*-value <0.05 were marked in green (*N* = 4). **b** Tables displaying the 15 most induced or reduced expressed genes, respectively, indicated in Supplementary Fig 3 by red squares. Relative gene expression is indicated by FPKM values (fragments per kilobase of exon per million fragments mapped)

To examine which target genes are differently transcribed in podocytes after Hippo pathway activation, we used LATS WT overexpressing cells for a transcriptome analysis by next-generation sequencing. In order to identify early differences in gene transcription and avoid secondary effects of apoptotic processes, we analyzed the transcriptome of cells in which LATS overexpression was induced for 8 h compared to not induced podocytes of the same cell line (for time dependency of LATS2 induction, see Supplementary Fig. 2). Overall 25.459 genes with an altered expression could be identified, of which 1428 turned out to be significantly changed with *q*-value <0.05 (Fig. 5a, Supplementary Table 1). As expected, the LATS2 mRNA level was increased most and was ignored during further analysis. The DEGs from our transcriptome analysis could be divided into 749 decreased and 679 increased genes (Supplementary Fig. 3). In Fig. 5b, the top 15 regulated genes are listed. Among the most downregulated genes, previously described YAP target genes like connective tissue growth factor, ankyrin repeat domain 1 and angiomotin-like 2 were found, validating our approach.

**Fig. 6:**
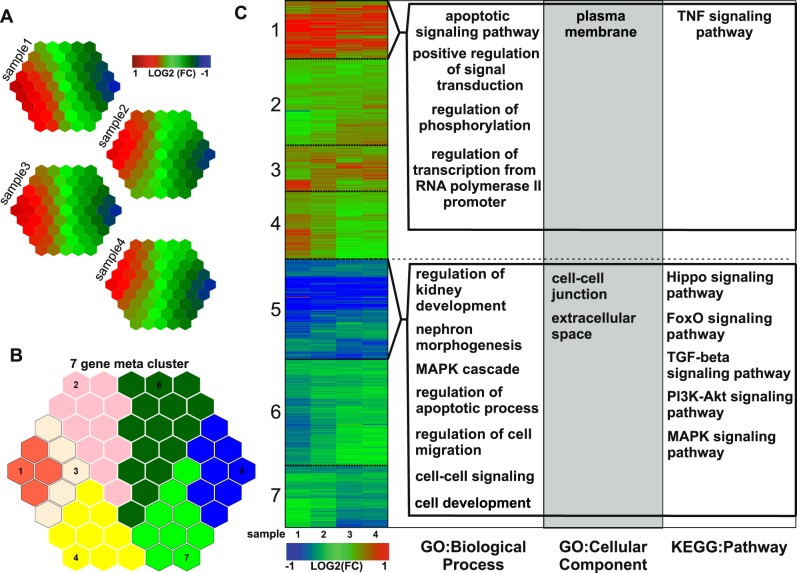
Clustering and functional annotation enrichment analysis of the differentially expressed genes. **a** Analysis of the transcriptional changed genes from the RNA sequencing experiment measured by means of a supra-hexagonal map. Sample specific clustering and visualization depending on differential expression patterns mapped onto the same or nearby regions in the map. **b** Subdivision and illustration of seven gene meta-clusters from all samples. **c** Visualization of the differential gene expression of all 1428 significantly changed genes by a fold change (FC) based heatmap divided in the seven gene meta-clusters. Clusters 1 and 5 were evaluated by annotation enrichment analysis performed by DAVID Bioinformatics Resources^40^ using the Gene Ontology (GO) terms—biological process and cellular component—as well as KEGG Pathways

Subsequently, the transcriptional changes of all four sample pairs were analyzed using a supra-hexagonal map in which genes with similar data pattern are attached to the same or nearby nodes in the map (Fig. 6a)^16^. A further topology-preserving partitioning operation could cluster the DEGs of these four sample pairs into seven gene meta-clusters of highly similar regulation patterns. The seven clusters are separated into: highly upregulated (cluster 1), upregulated (clusters 2–4), downregulated (clusters 6 and 7), and highly downregulated (cluster 5) (Fig. 6b, Supplementary Table 1). The DEGs of each cluster were used separately to perform functional annotation enrichment analyses on the Gene Ontology (GO) terms—biological processes and cellular components—as well as the Kyoto Encyclopedia of Genes and Genomes (KEGG) pathways. The main findings of the highly regulated clusters 1 and 5 are shown in Fig. 6c (the whole meta-analysis in Supplementary Fig. 4). The combined color coded heatmap indicates the transcriptional regulation of the corresponding genes.

As expected from our previous experiments, the Hippo pathway induced by LATS overexpression induces apoptosis. Clusters 1 and 4 include 56 significantly enhanced genes related to apoptotic regulation, while 50 transcriptional highly reduced genes from cluster 5 can be associated to anti-apoptotic functions (Supplementary Table 2). Additionally, an increased LATS activity obviously led to a downregulation of genes connected to the Hippo pathway (clusters 5 + 6), which normally regulate Hippo activity upstream of LATS, like neurofibromin 2 or KIBRA (Supplementary Table 3). Additionally several genes from non-canonical Hippo pathway regulation mechanisms like TGFß- and WNT-signaling pathways (TGFB2, BMP4, WNT2B) were transcriptionally reduced after LATS overexpression. This suggests a feedback mechanism to diminish Hippo pathway activity in consequence of YAP inactivation. The most interesting result from the screen was the influence of Hippo pathway activation on several processes concerning podocyte integrity. A lot of the significantly regulated proteins are known to play a role in cell–cell junctions or in the extracellular space, which in the case of podocytes concerns the GBM, or in cell migration (Supplementary Table 4). These results interconnect the Hippo pathway activation with the ability of podocytes to claim their place in the cellular network.

### Common transcriptional changes in FSGS and Hippo signaling activation may regulate podocyte epithelial integrity

**Fig. 7:**
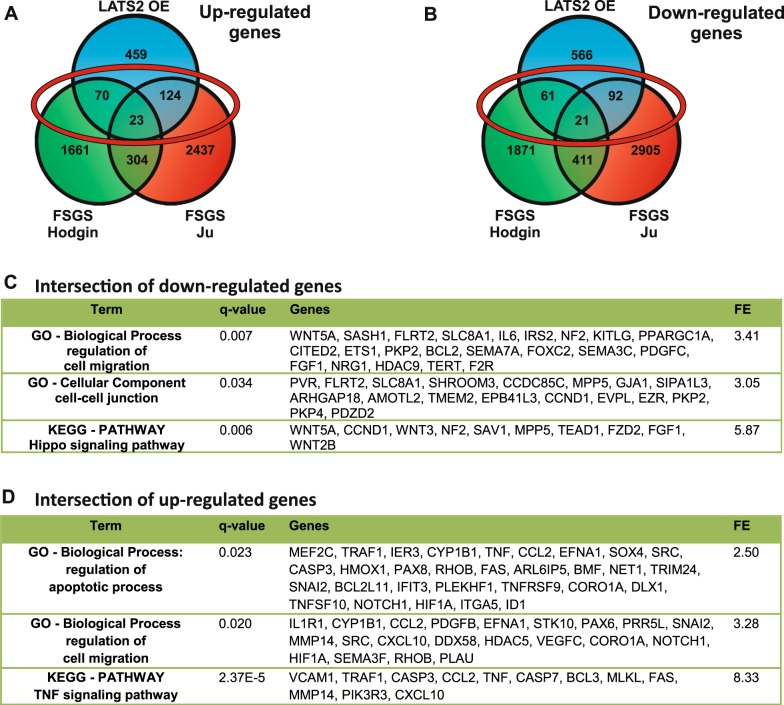
Comparison of transcriptional changes in Hippo pathway activated podocytes and glomeruli of FSGS patients. **a** Venn diagrams comparing downregulated or **b** upregulated genes from RNA sequencing of LATS2-overexpressing podocytes (LATS2) and glomeruli of FSGS patients from Hodgin et al.^27^ (FSGS Hodgin) and Ju et al.^28^ (FSGS Ju) to identify commonly regulated genes. **c** Functional annotation enrichment analysis from the intersection of downregulated or **d** upregulated genes in LATS2-overexpressing podocytes and in glomeruli of FSGS patients from Hodgin^27^ or Ju^28^ (intersections indicated by red circles in **a** and **b**)

The observations that (i) podocyte-specific YAP deletion in mice results in FSGS and (ii) the amount of p-YAP increases in human FSGS give a hint that Hippo pathway activation is linked to FSGS development^8^. Therefore, we compared the dataset from our transcriptome analysis with the data from two independent studies of transcriptional regulation in glomeruli from human FSGS patients (transcriptomic datasets from Hodgin et al.^27^ and Ju et al.^28^) obtained from the Nephroseq database (www.nephroseq.org). The intersection of these two sets of patient data is surprisingly small, probably due to the natural inconsistency of patient samples. Therefore, we initially analyzed the intersection of 677 genes, which were regulated in LATS2-overexpressing podocytes and one of the datasets from FSGS glomeruli independent from the direction of regulation (Supplementary Fig. 5). The analysis of this reduced number of genes from the intersection still resulted in main parts in the same significantly enriched GO terms and KEGG pathways we had found before in the analysis of LATS2 overexpression (Fig. 6). The most important results were the regulation of genes influencing apoptosis, the Hippo signaling pathway, PI3K-AKT signaling pathway, and TNF signaling pathway, as well as regulation of cell–cell junctions and cell migration. In a further comparison, up and downregulated genes of all three datasets were evaluated separately (Fig. 7). Regarding the intersection of downregulated genes found in LATS2 overexpression and at least one FSGS dataset, the only significantly enriched KEGG pathway is the Hippo signaling pathway, which confirms a downregulation especially of these genes. Significantly enriched GO terms concerned the regulation of cell migration and cell–cell junctions, which gives a hint that Hippo pathway activity in FSGS influences podocyte integrity. The intersection of upregulated genes confirms the induction of apoptotic processes in FSGS and again influence on the regulation of cell migration and TNF signaling pathway.

## Discussion

The cellular localization of YAP in podocytes in vivo is still a subject of discussion^5,17^. Analyzing human and rat kidney slices, we could confirm a predominant nuclear staining of YAP in podocytes and a co-localization with the podocyte-specific marker protein WT1. This observation supports the notion that nuclear YAP localization in cultured podocytes indeed reflects the in vivo situation and can be used as an appropriate model system. Our data presented here further underline the mandatory function of nuclear YAP as a pro-survival factor for podocytes. An activation of the Hippo pathway connected with a nuclear export of YAP in podocytes led to the induction of apoptosis. This process was induced in various ways: a decrease of substrate rigidity (Fig. 1), by incubation with the SRC inhibitor dasatinib (Fig. 2), or by overexpression of the upstream Hippo pathway members LATS2 (Figs. 3 and 4) or KIBRA^6^. Interestingly, similar effects on nuclear YAP and the induction of apoptosis were observed in podocytes treated with high glucose, lipopolysaccharides, or adriamycin^29^. Therefore, we conclude that nuclear YAP is crucial for the podocyte functionality.

Beside canonic Hippo signaling, the assembly and dynamics of actin stress fibers obviously have an important impact on YAP activity in podocytes. Cytoskeletal tension was shown to regulate YAP nuclear localization especially under pathological conditions^18^. We could show before that the disruption of the actin cytoskeleton with latrunculin B leads to an activation of the Hippo pathway and a loss of nuclear YAP in podocytes in vitro^6^. The decrease of substrate rigidity (Fig. 1) as well as the treatment with dasatinib (Fig. 2) besides Hippo pathway activity influenced the actin cytoskeleton. Interestingly, an intact actin cytoskeleton seems to be crucial for glomerular functionality. Mutations in proteins influencing the actin dynamics (e.g., α-actinin-4, INF2, CDC42) are known to cause kidney injuries including nephrotic syndrome or FSGS^31–34^. On the other side, FSGS was recently linked to an activation of the Hippo pathway, confirmed by an increased phosphorylation of YAP and an increased expression of the Hippo pathway upstream regulator KIBRA^8^. This may indicate a direct connection between Hippo pathway activity, functionality of the actin cytoskeleton, and kidney injuries. Using our podocyte cell culture model, we could show that dasatinib treatment beside Hippo pathway activation results in the induction of apoptosis (Fig. 2). The expression of permanently nuclear YAP could diminish this apoptosis induction significantly, which confirmed the participation of the Hippo signaling pathway. Interestingly, treatment of leukemia patients with dasatinib occasionally leads to impaired kidney function and the development of nephrotic-range proteinuria. Microscopic analyses of biopsies from dasatinib-treated patients revealed thinning of the GBM as well as focally effaced or fused foot processes as well as sclerosis^22,34^. Also diabetic glomerulosclerosis goes along with changes in the GBM and loss of podocytes^35^. In conclusion, these observations raise the question if an activation of the Hippo pathway in podocytes is a general consequence of kidney injury and if nuclear exclusion of YAP commonly impairs podocyte functionality and homeostasis.

The imperative of nuclear YAP for podocytes suggests a cell type-specific transcription program controlled through the Hippo pathway and the actin cytoskeleton. To proof this hypothesis on the messenger RNA level, we first tried a YAP knockout in cultured podocytes, which were not viable, probably due to the importance of YAP for podocytes. Inducible YAP knockdown cells, however, displayed only minor effects on gene transcription and apoptosis, probably due to the nuclear localization and activity of the remaining YAP (Supplementary Fig. 6). Nuclear import of YAP is prevented by LATS-mediated phosphorylation at Ser127. LATS itself must be phosphorylated at Thr1041 to become activated. The comparison of overexpressed wild-type LATS2 with the two phospho-mutants demonstrated that overexpression of wild-type LATS2 was sufficient to exclude YAP from the nucleus and induce apoptosis. In order to determine the immediate effects of Hippo pathway activation and to minimize secondary effects of a proceeding apoptosis, we performed a transcriptome analysis of samples at an early point of LATS2 overexpression (Supplementary Fig. 2). It must be mentioned that following this setup transcriptional effects of the two strongly related co-transcription factors YAP and TAZ as well as potential further effects cannot be distinguished.

Our data from the transcriptome analysis indicated a significant upregulation of a large set of apoptotic genes (Fig. 6). The findings nicely represent the huge variety of apoptotic processes induced by the loss of nuclear YAP in podocytes. Interestingly, an excessive Hippo pathway activation in podocytes due to overexpression of LATS2 led to a downregulation of other upstream Hippo pathway components in terms of a negative feedback loop (Fig. 6). This kind of regulative feedback loop could already be described for LATS activation in other cell types^36^. An important observation from the transcriptome analysis was the impact of Hippo pathway activation on the expression of genes involved in cell–cell junctions and composition of extracellular space as well as cell migration. This suggests that Hippo pathway activation influences the integrity of in vivo tightly interlocked podocytes. It can therefore be hypothesized that not only a disturbance of the actin cytoskeleton may activate the Hippo pathway, but also that an extraordinary activation of the Hippo pathway may be causative for podocyte damage.

Podocyte-specific deletion of YAP in mice causes podocyte apoptosis, resulting in proteinuria and FSGS^5^. On the other hand, FSGS seems to go along with Hippo pathway activation connected to an increased KIBRA expression and YAP phosphorylation in human^8^. Furthermore, KIBRA deletion in mice is protective against podocyte foot process effacement after protamine sulfate perfusion^8^. Therefore, not ignoring the fact that two different systems were compared, we matched results from LATS-overexpressing cultured podocytes with those of injured glomeruli containing a diversity of cell types, which additionally underlay the influences of a whole organism^27,28^. Interestingly, a multitude of consistent regulations could be observed. The intersection of up and downregulated genes resulted in the regulation of genes influencing apoptosis, the Hippo signaling pathway, as well as the TNF signaling pathway. Interestingly, we observed a significant regulation of genes influencing cell junction formation as well as cell migration in the intersection of the datasets from LATS2-overexpressing podocytes and glomeruli from FSGS patients. It is therefore tempting to speculate that these results link Hippo pathway activation in podocytes to the regulation of cellular junction and migration in vivo, which might be a fundamental mechanism of glomerular sclerosis and loss of renal function. Several facts underline this hypothesis. For example, a knockdown of the Hippo pathway upstream member KIBRA in podocytes reduces their ability for directed migration^37^. KIBRA was identified as an interaction partner of the Crumbs complex component PATJ. The polarity complex Crumbs/Pals1 (MPP5)/PATJ is localized to the apical membrane of polarized cells and its function is associated with tight junction formation. The complex is known to influence the Hippo pathway in two ways. First, the formation of the Crumbs complex directly leads to activation of LATS kinase^38^. On the other hand, the Crumbs complex binds YAP/TAZ, recruits them to the membrane, and therefore prevents nuclear localization of these co-transcription factors^38^. In agreement with that, we could recently show that a knockdown of Pals1 in tubular kidney cells results in an increased nuclear localization of YAP/TAZ and in a delay of junction formation^39^. As the knockdown of Pals1 in the whole mouse nephron led to cyst formation and Hippo pathway inactivation, we hypothesized that the integrity of tight junctions is a crucial factor in the control of Hippo signaling.

In conclusion, we show that sequestration of YAP into the cytoplasm in podocytes results in the induction of apoptosis. The upregulation of apoptotic genes was confirmed in a RNA sequencing analysis of LATS2-overexpressing podocytes. Interestingly, we demonstrate consistent transcriptional changes of these apoptotic genes in a comparison with the datasets from glomeruli of FSGS patients. This comparison further discovered a potential interplay between Hippo pathway regulation and cell migration and junction formation in vivo and in vitro, which in case of abnormal regulation might induce glomerular sclerosis and finally loss of renal function. Therefore, our work highlights the importance of YAP for normal podocyte function and gives a promising outlook on the possibility of therapeutic manipulation of Hippo signaling in podocytes.

## Electronic supplementary material

Supplemental Table 1

Supplemental Material
