# Supplementary material for: Nuclear YAP localization as a key regulator of podocyte function
Source: Cell Death Dis. 2018 Aug 28;9(9):850. doi: 10.1038/s41419-018-0878-1 (PMC6113334; doi:10.1038/s41419-018-0878-1)
Supplement: Supplementary file 2 — Supplemental Material [file 41419_2018_878_MOESM2_ESM.pdf]

Suppl.Fig.1:

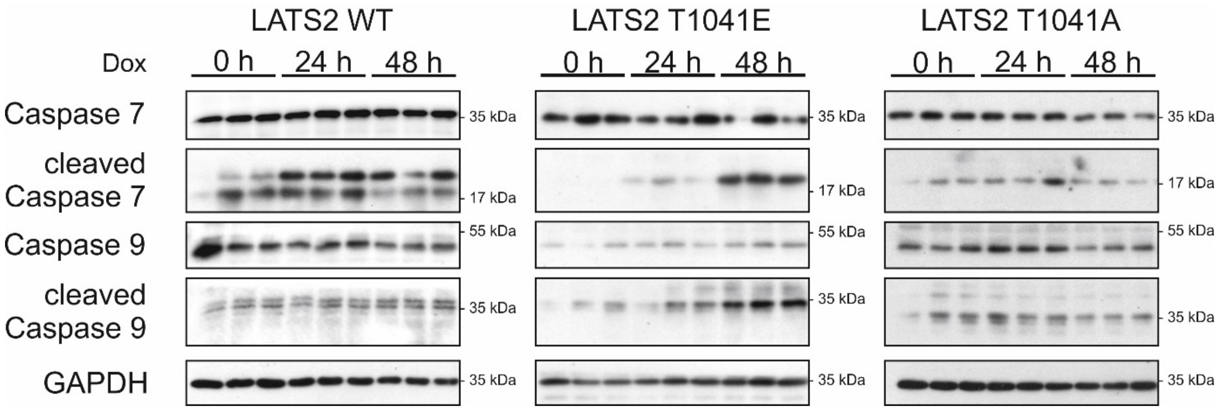

Suppl.Fig.2: Western blot analyses of lysates from LATS2 wild type (LATS2 WT), permanently active (LATS2 T1041E) and permanently inactive LATS2 (LATS2 T1041A) overexpressing podocyte cell lines in triplicates at 0, 24 and 48 hours doxycycline induction to determine cleavage of apoptotic marker proteins Caspase 7 and 9. GAPDH serves as loading control.

Suppl.Fig.2:

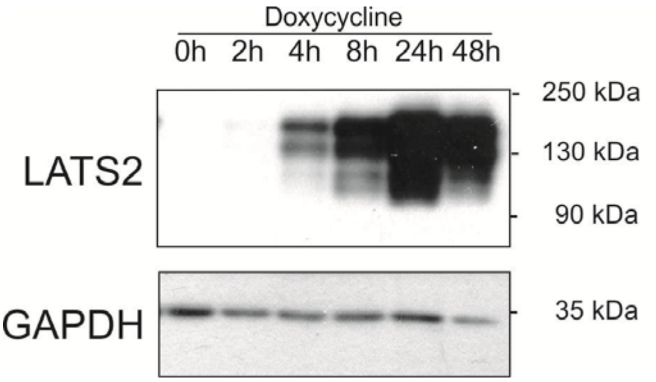

Suppl.Figure 3: Western blot analysis of LATS2 in a doxycycline inducible human podocyte LATS2 WT overexpressing cell line at 0, 2, 4, 8, 24 and 48 h after doxycycline induction. GAPDH serves as loading control.

Suppl.Fig.3:

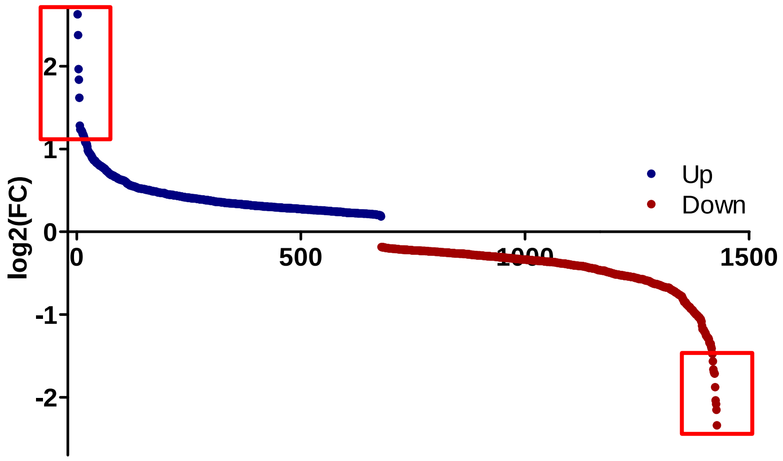

Suppl.Figure 4: Regulation in (base 2) logarithmic fold change of the 1428 significantly changed genes with FPKM values > 1 from RNA sequencing analysis in Figure 5. Genes are displayed separated in 679 increased expressed (“Up”, blue) and 749 decreased expressed genes (“down”, red). Top 15 up- and down-regulated genes (indicated by red square) are displayed in tables in Figure 5 B, respectively.

Suppl.Fig.4:

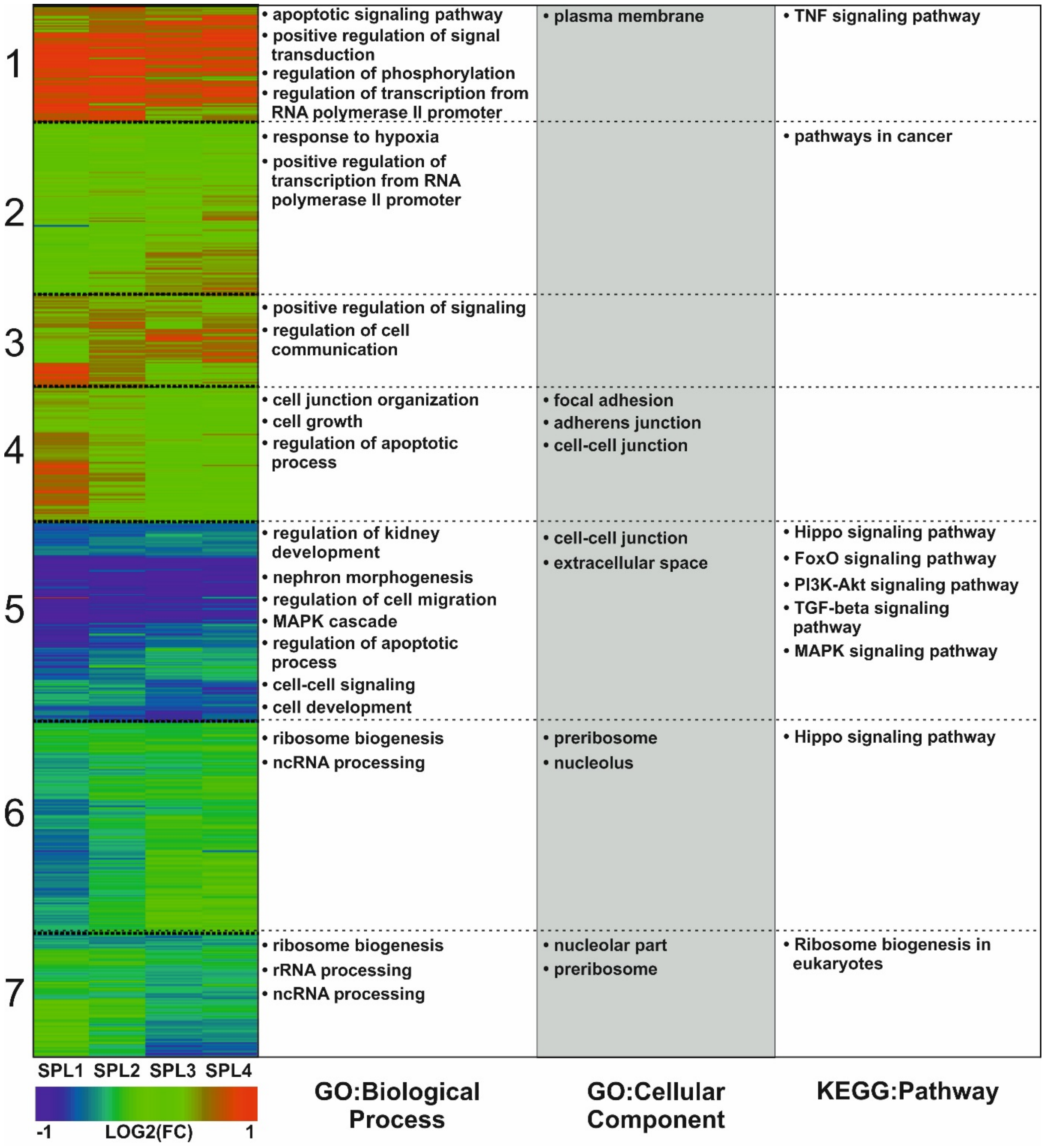

Suppl.Figure 5: Tabular visualization of a fold change (FC) based heat map displaying differential gene expression of all 1428 significantly changed genes separated into 7 gene meta clusters evaluated by annotation enrichment analyses. Gene Ontology (GO) terms of Biological Process and Cellular Component, as well as KEGG Pathways were used for enrichment analysis performed by DAVID Bioinformatics Resources. SPL1-4: Sample 1-4.

Suppl.Table 2:

Suppl.Table2: **Transcriptional regulation of apoptosis related gene sets.**
GO Term - BP = Gene Ontology – Biological Process; FE=Fold Enrichment.

GO Term - BP	#genes	cluster	q-value	genes	FE
**apoptotic signaling pathway**	21	1	3.71E-04	TRAF1, CSF2, CEBPB, CYP1B1, MKNK2, IL6R, CX3CL1, SNAI1, BCL2L11, DDIT4, HIC1, PPIF, TNFRSF9, TNFSF10, CDKN1A, HIF1A, ATF3, BBC3, HMOX1, BCL3, BMF	4.23
**regulation of apoptotic process**	35	4	3.04E-03	IER3, CCL2, THRA, CBX4, PML, NFKBIA, SOX4, HSPA1A, OSGIN1, HSPA1B, SFN, SRC, VDR, TNFRSF1A, SQSTM1, PLEKHG5, DDAH2, PHLDA3, HIP1, DAB2IP, RARG, ABR, ARHGEF16, CD40, NOTCH1, CORO1A, KDM2B, LRP1, ITGA5, ID1, VEGFA, ID3, GRK5, SLC9A1, PNMA1	2.51
**regulation of apoptotic process**	50	5	1.30E-06	DLC1, SH3RF1, NOG, HNF1B, NUAK2, GDF6, EDN1, MITF, ANKRD1, PAWR, GDNF, TGFB2, CITED2, DAB2, BDNF, TNFRSF11B, TIAM2, CTGF, NRG1, FOSL1, MYC, CYR61, EGR1, IRS2, SGK1, TLE1, PPARGC1A, FMN2, INHBA, AMIGO2, ADM, F3, IL12A, FOXC2, GADD45B, GADD45A, NGF, TFAP4, BCL2L1, BCL2, BMP4, ACTC1, IL6, NTF3, MALT1, DUSP1, PLK2, ETS1, CD274, IGFBP3	2.66

Suppl.Table 3:

Suppl.Table3: **Transcriptional regulation of genes related to the KEGG Pathway - Hippo signaling pathway.**

KEGG = Kyoto Encyclopedia of Genes and Genomes Pathways**;** **FE** = Fold Enrichment.

KEGG	#genes	cluster	q-value	genes	FE
**Hippo signaling pathway**	13	5	3.21E-04	AJUBA, BMP4, AMH, FRMD6, NF2, CTGF, GDF6, MPP5, WWC1, WTIP, MYC, WNT2B, TGFB2	5.72
**Hippo signaling pathway**	9	6	2.00E-02	WNT5A, YWHAG, WNT3, SAV1, SERPINE1, TEAD1, FGF1, WWTR1, FZD7	4.11

Suppl. Table 4:

Suppl.Table 4: **Transcriptional regulation of gene sets concerning podocyte integrity.**
GO = Gene Ontology; CC = Cellular Component; BP = Biological Process; FE=Fold Enrichment.

GO Term	#genes	cluster	q-value	genes	FE
**CC - focal adhesion**	14	4	5.21E-03	LIMK1, PIP5K1C, HSPA1A, HSPA1B, ASAP3, MMP14, JUP, TNS3, LRP1, RAC2, ITGA5, GRK5, SLC9A1, PNMA1	3.66
**CC - adherens junction**	23	4	4.83E-04	DAB2IP, LIMK1, BAIAP2, ARHGEF16, PIP5K1C, HSPA1A, CAMSAP3, HSPA1B, SFN, ASAP3, MMP14, SRC, JUP, TNS3, NOTCH1, LRP1, RAC2, ITGA5, PKP3, TNK2, GRK5, SLC9A1, PNMA1	3.39
**CC - cell-cell junction**	19	4	2.54E-02	MICALL2, DAB2IP, BAIAP2, FGFRL1, VANGL2, SRC, JUP, ARHGEF16, HSPA1A, CAMSAP3, HSPA1B, SFN, SV2A, CORO1A, ITGA5, PKP3, PLEKHG5, FHOD1, SLC9A1	3.02
**CC - cell-cell junction**	22	5	1.46E-02	KAZN, FLRT2, SLC8A1, TRPC4, PPME1, SHROOM3, CCDC85C, MPP5, GJA1, ASAP1, OXTR, WTIP, AMOTL2, EPHA2, TMEM2, AJUBA, FRMD6, CGN, PKP2, CLDN1, PDZD2, PHLDB2	2.56
**CC - extracellular space**	37	5	3.74E-02	ACTBL2, CXCL1, FGF5, NOG, EDN2, GDF6, EDN1, CXCL8, TGFB2, LIF, KISS1, TNFRSF11B, CTGF, NRG1, FGF2, BMP4, FLRT2, CPA4, IL6, ACTC1, MDGA1, KRT34, TINAGL1, WNT2B, INHBA, AMH, DKK1, ADM, CST6, F3, IL12A, FJX1, HBEGF, ADAMTS3, IGFBP3, ADAMTS5, VLDLR	1.92
**BP - regulation of cell migration**	43	5	5.32E-13	CXCL1, DLC1, NOG, EDN2, EDN1, CXCL8, STARD13, ETS1, TGFB2, CITED2, DAB2, S1PR1, BCL2, NRG1, MYC, FGF2, CYR61, BMP4, FLRT2, SASH1, SLC8A1, IL6, PKP2, GTPBP4, SGK1, IRS2, NTF3, NF2, NEXN, PPARGC1A, FAM60A, EPHA2, C5ORF30, AJUBA, F3, CD274, IL12A, HBEGF, FOXC2, HAS2, SMURF2, IGFBP3, PHLDB2	4.68

Suppl.Fig.5:

A

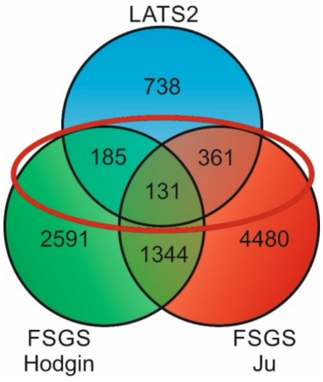

B

Intersection of genes regulated in LATS2 overexpressing podocytes and glomeruli of FSGS patients (datasets of Ju or Hodgin)

Term	q-value	Genes	FE
**GO – Biological Process: regulation of apoptotic process**	2.35E-14	MEF2C, THRA, PNMA2, ZAK, EFNA1, GDF5, CBX4, LPAR1, GDNF, IL11, CITED2, BDNF, TIAM2, SERPINE1, FAS, DDAH2, NET1, IRS2, CD40, PPARGC1A, BCL2L11, HIF1A, ARRB1, TNFAIP8, VEGFA, RIPK2, MAPK8, NGF, TRAF1, WNT5A, CAV1, CYP1B1, CCL2, TFAP4, MCL1, SOX4, NFKBIA, CX3CL1, BCL2L1, PTRH2, SRC, VDR, NDRG1, ARL6IP5, CFLAR, TCF7, NOTCH1, CORO1A, TNFSF10, DLX1, CDKN1B, ATF3, PLK2, UCP2, ITGA5, ETS1, PTTG1IP, ABL1, DNAJB6, IER3, NUAK2, PAWR, GLI3, TNFRSF11B, CASP3, CLCF1, BOK, CASP7, HMOX1, PAX8, RHOB, NRG1, TERT, DHCR24, EGR1, SGK2, ARHGEF16, TLE1, DAPK1, SERPINB9, TNFRSF9, BTG2, ADM, FOXC2, G0S2, GADD45B, GADD45A, TNFRSF21, CSF2, TNF, SAV1, FHL2, KITLG, IVNS1ABP, TSC22D3, SQSTM1, BCL2, BCL3, NEFL, IL6, CEBPB, TRIM24, STAT1, SNAI2, ISL1, SIRT1, PLAC8, PLEKHF1, IFIT3, NUPR1, DUSP1, ID1, PPP1R13B, ID3, GDF15, IGFBP3, F2R, BMPR1A	2.31
**GO – Biological Process: regulation of cell motility**	1.01E-11	NRP2, PLXNA3, PDGFB, EFNA1, JAG2, PAX6, ARHGAP18, LPAR1, CITED2, CXCL10, SEMA7A, SEMA3F, HMOX1, GAB1, SERPINE1, RHOB, SEMA3C, PDGFC, ATOH8, FGF1, NRG1, TERT, PTPRJ, GTPBP4, IRS2, IL6R, MMP14, PPARGC1A, VEGFC, HIF1A, VEGFA, FOXC2, WNT5A, CXCL1, IL1R1, CCL2, CYP1B1, PPP2R3A, TNF, ABHD6, STK10, CSF1, KITLG, CX3CL1, SRC, RAC2, NDRG4, BCL2, PLXND1, FLRT2, SASH1, IL6, SLC8A1, NF2, PODXL, PRR5L, SNAI2, EPHA2, DDX58, HDAC5, CORO1C, NOTCH1, CORO1A, ITGA5, PKP2, ETS1, HBEGF, SMURF2, HDAC9, ABL1, IGFBP3, ABL2, PLAU, F2R, BMPR1A	2.80

**GO – Biological Process: regulation of cell migration**	2.63E-11	NRP2, PLXNA3, PDGFB, EFNA1, JAG2, PAX6, LPAR1, CXCL10, CITED2, SEMA7A, SEMA3F, HMOX1, GAB1, SERPINE1, RHOB, SEMA3C, PDGFC, ATOH8, FGF1, NRG1, TERT, PTPRJ, GTPBP4, IRS2, IL6R, MMP14, PPARGC1A, VEGFC, HIF1A, VEGFA, FOXC2, CXCL1, WNT5A, IL1R1, CCL2, CYP1B1, TNF, ABHD6, STK10, CSF1, KITLG, CX3CL1, SRC, RAC2, NDRG4, BCL2, PLXND1, FLRT2, SASH1, IL6, SLC8A1, NF2, PODXL, PRR5L, SNAI2, EPHA2, DDX58, HDAC5, CORO1C, NOTCH1, CORO1A, ITGA5, PKP2, ETS1, HBEGF, SMURF2, HDAC9, IGFBP3, PLAU, F2R, BMPR1A	2.84
**GO – Biological Process: cell adhesion**	1.81E-7	PVR, MTSS1, ZC3HAV1, EFNA1, FSTL3, JAG2, CITED2, KIFC3, TGFBI, SERPINE1, FAS, NET1, PTPRJ, GTPBP4, EFNB1, BYSL, BAIAP2, SOCS5, BCL2L11, VEGFC, VEGFA, RIPK2, GBP1, WNT5A, CAV1, CYP1B1, SCN1B, CCL2, STK10, NEDD9, ASAP1, SOX4, CX3CL1, FXYD5, SRC, CDC42EP1, RAC2, DMD, NDRG1, SPP1, TCF7, PODXL, PCDH10, TINAGL1, EPHA2, CORO1C, EPHA4, NOTCH1, CORO1A, CD55, ETS1, NEDD4, PLEKHA7, PKP2, ITGA5, PKP3, CD58, PKP4, PDZD2, ABL1, ABL2, PLEKHA2, DNAJB6, PLAU, NRP2, LIMA1, CLDN4, PIP5K1C, ARHGAP18, PIP5K1A, PAWR, GLI3, MMP24, RHOB, CNTNAP1, NRG1, VASN, EGR1, ARHGEF16, PCDH7, MMP14, SLC7A11, CD83, EVPL, CLDN1, FOXC2, ZFPM1, TNFRSF21, TNF, TNC, CSF1, KITLG, APBB1IP, SCARF2, VCAM1, EZR, COL7A1, C1QTNF1, BCL2, BCL3, COL8A1, APBA1, ZBTB7B, MICALL2, HAPLN1, FLRT2, IL6, CEBPB, NF2, STAT1, SNAI2, FZD5, TMEM2, ID1, IRF1, CDH11	1.87
**GO – Biological Process: regulation of MAPK cascade**	1.15E-6	MEF2C, ZAK, PDGFB, EFNA1, GDF5, CSPG4, LPAR1, IL11, KISS1, TNFRSF11B, DIRAS2, MAP3K4, SEMA7A, GAB1, PDGFC, FAS, NRG1, FGF1, IRAK2, PTPRJ, IL6R, CD40, ADRB2, DACT1, ARRB1, VEGFA, RIPK2, GADD45B, GADD45A, GBP1, NGF, WNT5A, CAV1, CCL2, TNF, DUSP10, TRIB3, KITLG, CX3CL1, SRC, LIF, EZR, DUSP14, NDRG4, DMD, C1QTNF1, ARL6IP5, SASH1, IL6, NF2, FZD5, MID1, EPHA2, EPHA4, ATF3, DUSP1, ID1, GDF15, ABL1, IGFBP3, F2R	2.47
**GO – Biological Process: cell junction organization**	0.003	PVR, CAV1, TNF, PIP5K1C, GJA1, PIP5K1A, SRC, KIFC3, BCL2, CNTNAP1, PTPRJ, MICALL2, NF2, MPP5, PTPN13, FZD5, SNAI2, MMP14, CORO1C, EPB41L3, PLEKHA7, PKP2, ITGA5, PKP3, VEGFA, PKP4, CLDN1, CDH11	3.09
**GO – Biological Process: extracellular matrix organization**	0.036	PHLDB1, TNF, CYP1B1, ELF3, PDGFB, LUM, TNC, MMP2, VCAM1, TNFRSF11B, COL7A1, TGFBI, SERPINE1, BCL3, COL8A1, COL11A1, SPP1, SH3PXD2B, HAPLN1, FLRT2, MMP14, COL5A2,	2.55

		NOTCH1, ETS1, ITGA5, FOXC2, MFAP2, ADAMTS3, ABL1, ADAMTS5, DNAJB6	
**GO – Cellular component: adherens junction**	5.64E-5	PVR, KIF23, LIMA1, ZC3HAV1, CSPG4, GJA1, PDLIM2, ARHGAP18, PIP5K1C, PIP5K1A, KIFC3, STARD8, RHOB, VASN, BAIAP2, ARHGEF16, MMP14, ARHGAP26, EVPL, SH3KBP1, CAV1, SHROOM3, TNC, FHL2, NEDD9, ASAP1, ASAP3, APBB1IP, SRC, SCARF2, EZR, CDC42EP1, RAC2, NDRG1, AFAP1, FLRT2, DIXDC1, NF2, FZD2, STAT1, EPHA2, TMEM2, EPHA5, CORO1C, NOTCH1, ITGA5, PKP2, PLEKHA7, PKP3, IRF2, SYNM, MARCKS, TNK2, PLAU	2.22
**GO – Cellular component: cell-cell junction**	0.003	PVR, LIMA1, SHROOM3, SCN1B, CCDC85C, CLDN4, ZC3HAV1, GJA1, ARHGAP18, ASAP1, AMOTL2, SRC, KIFC3, EZR, CDC42EP1, DLG3, NDRG1, CDC42EP4, MICALL2, PTPRJ, VASN, FLRT2, SLC8A1, BAIAP2, PODXL, MPP5, ARHGEF16, SIPA1L3, FZD5, STAT1, EPHA2, TMEM2, DDX58, EPB41L3, CCND1, CORO1A, EVPL, ITGA5, PKP2, PLEKHA7, PKP3, PKP4, CLDN1, SH3KBP1, PMP22, PDZD2, FHOD1	2.08
**GO – Cellular component: focal adhesion**	0.005	PVR, KIF23, LIMA1, CAV1, TNC, CSPG4, FHL2, PDLIM2, NEDD9, PIP5K1C, GJA1, PIP5K1A, ASAP3, APBB1IP, SCARF2, CDC42EP1, EZR, RAC2, STARD8, RHOB, AFAP1, DIXDC1, FLRT2, FZD2, MMP14, EPHA2, ARHGAP26, CORO1C, ITGA5, SH3KBP1, IRF2, MARCKS, PLAU	2.40
**KEGG – Pathway: TNF signaling pathway**	6.09E-8	CXCL1, TRAF1, CFLAR, CSF2, IL6, TNF, CCL2, CEBPB, CSF1, NFKBIA, CX3CL1, MMP14, JUNB, CXCL10, VCAM1, LIF, CASP3, CASP7, BCL3, MLKL, MAPK8, FAS, PIK3R3	5.18
**KEGG – Pathway: PI3K-Akt signaling pathway**	1.70E-4	FGF5, PPP2R3A, MCL1, PDGFB, PPP2R5B, EFNA1, CSF1, TNC, TLR2, KITLG, GNG11, LPAR2, BCL2L1, LPAR1, BCL2, PDGFC, FGF1, PIK3R3, COL11A1, SPP1, IL6, SGK2, IL6R, COL5A2, EPHA2, BCL2L11, DDIT4, VEGFC, CCND1, CDKN1B, LPAR5, ITGA5, VEGFA, EPOR, F2R, NGF	2.49
**KEGG – Pathway: Hippo signaling pathway**	0.012	WNT5A, TCF7, NF2, SAV1, GDF5, MPP5, TEAD1, FZD2, FZD5, SNAI2, WNT2B, CCND1, WNT3, ID1, TEAD4, SERPINE1, DLG3, FGF1, BMPR1A	3.00

Suppl.Figure 6: A) Venn diagrams comparing significant regulated genes from RNA-Sequencing of LATS2 overexpression podocytes (LATS2) to transcriptional changes in glomeruli of FSGS patients from Hodgin et al., 2010[20] (FSGS Hodgin)and Ju et al., 2013[21](FSGS Ju)to identify common regulated genes. B) Table displaying the results from annotation enrichment analysis of the intersections from LATS2 overexpression to FSGS Hodgin and FSGS Ju regulated genes indicated by red circle in A.

Suppl.Fig.6:

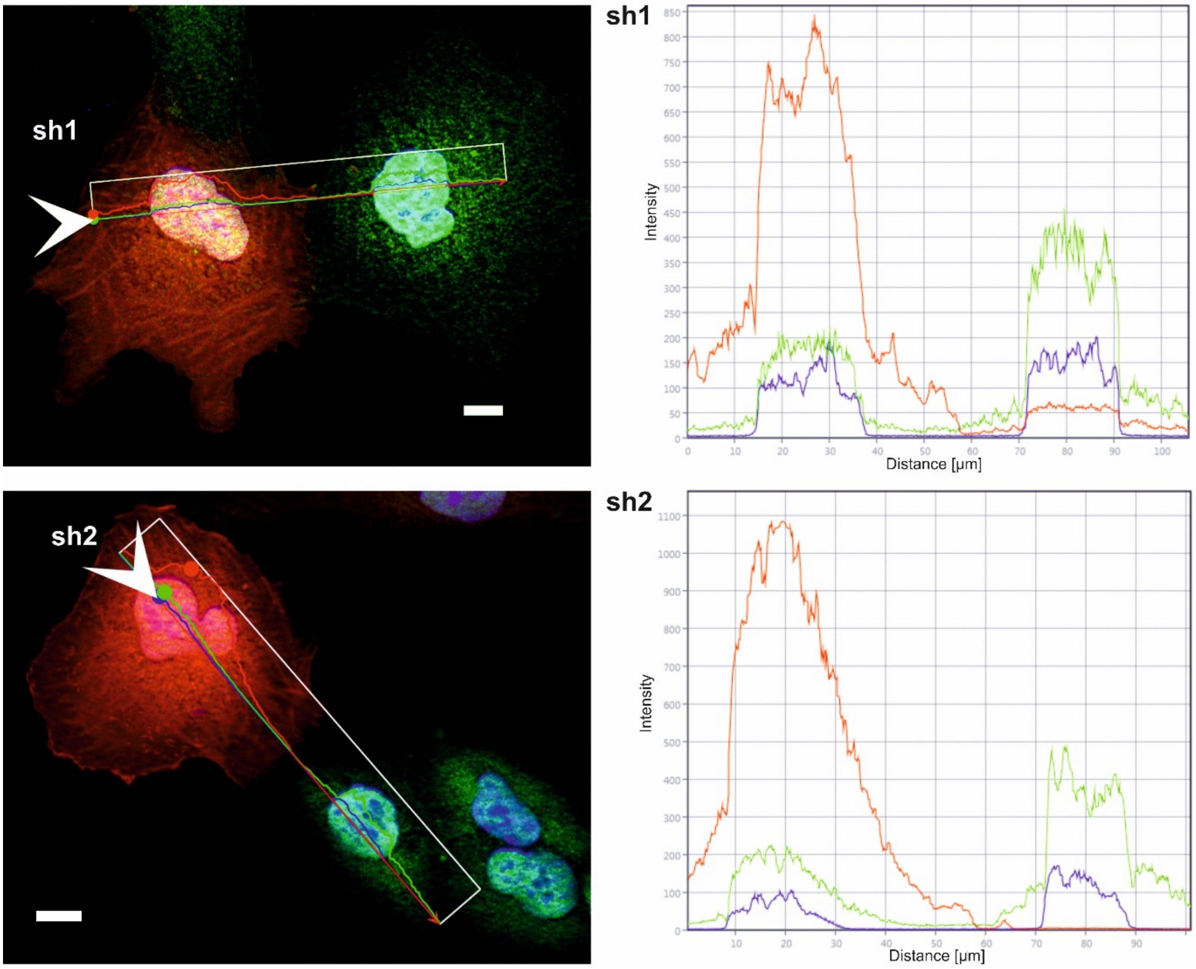

Suppl.Figure 7: YAP (green), RFP (red) and DAPI (blue) co-staining of doxycycline induced (120 h) YAP-knockdown cell lines co-expressing RFP and short hairpin RNA (sh1 or sh2). Comparison of the fluorescence intensity from cell couples with high versus low RFP/shRNA expression reveals reduction but nuclear localization of the remaining YAP. Scale bar=10 µm.

Suppl. Table 5:

Supp.Table 5: **Primer used for quantitative real time PCR.**

Target	Species	Direction	Sequence (5'-3')
**ANKRD1**	human	forward	TAGCGCCCGAGATAAGTTGC
**ANKRD1**	human	revers	GTCTGCCTCACAGGCGATAA
**CTGF**	human	forward	CACCCGGGTTACCAATGACA
**CTGF**	human	revers	GGATGCACTTTTTGCCCTTCTTA
**EDN1**	human	forward	CACAAAGGCAACAGACCGTG
**EDN1**	human	revers	GGTCTCCGACCTGGTTTGTC
**BCL2**	human	forward	GTCATGTGTGTGGAGAGCGT
**BCL2**	human	revers	GGGCCAAACTGAGCAGAGTC
**SGK1**	human	forward	TGGGCTACCTGCATTCACTG
**SGK1**	human	revers	GCTATAAAAAGGCGGCAGGC
**BCL2L1**	human	forward	GCCATCAATGGCAACCCATC
**BCL2L1**	human	revers	TCCACAAAAGTATCCTGTTCAAAGC
**BMF**	human	forward	GTTCCAACCAGAGGATGGGG
**BMF**	human	revers	CCGATAGCCAGCATTGCCATA
**GAPDH**	human	forward	CAAGCTCATTTCCTGGTATGAC
**GAPDH**	human	revers	GTGTGGTGGGGGACTGAGTGTGG
